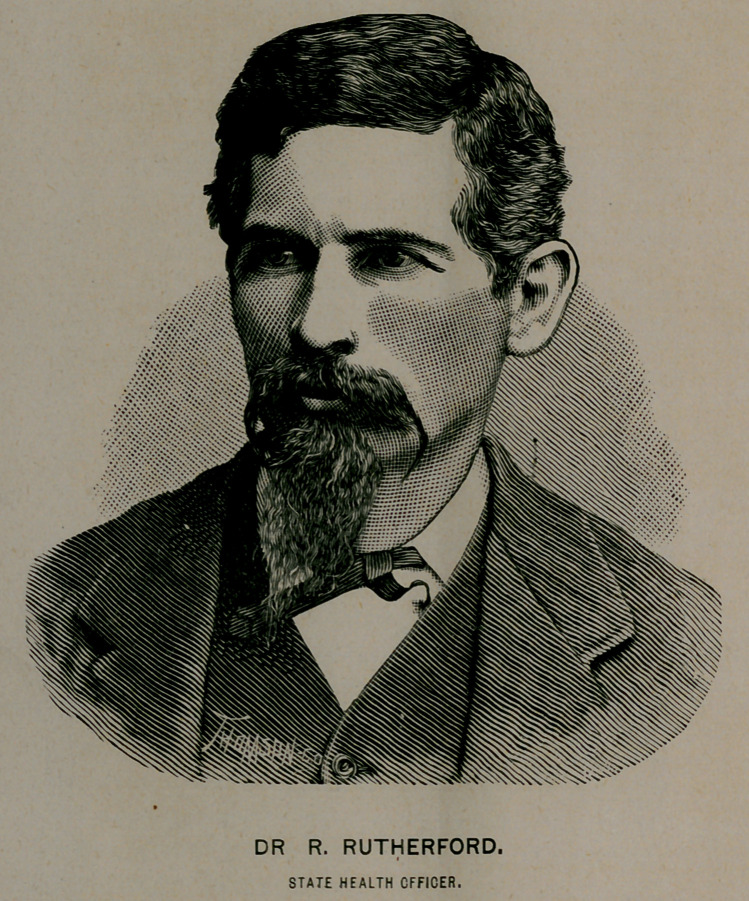# The New State Health Officer, Dr. R. Rutherford, with Portrait

**Published:** 1887-03

**Authors:** 


STATE HEALTH OFFICER.

### Biographical.

### THE NEW STATE HEALTH OFFICER OF TEXAS.

(SUBJECT OF ILLUSTRATION.)

Dr. Robert Rutherford, the newly appointed State Health Officer for Texas, is a son of Col. Vivian Rutherford, of Georgia, and was born in Columbus, in that State. He received an "old field school" education, and afterwards a collegiate course at the University of Georgia. His professional studies were pursued at Nashville, and at the University of New York. On the breaking out of the war he entered as a soldier in the Second Georgia Regiment, Nelson's Rangers, and was taken prisoner, and carried to Fort Delaware, Alton, Ill., where he was confined twelve months. The war over, he started to Mexico, but stopped in Wharton county, Texas, (1886) where he settled and engaged in the practice of medicine. Thence he removed to Brazos, and in 1871 removed to Houston, where he has resided continuously up to date. Dr. Rutherford was married in 1867 to Amanda Cardwell, sister of Col. John Cardwell, late of of Austin, but at present Diplomatic Agent and Consul General of the United States at Cairo, Egypt. His wife is a native of Lexington, Ga.

Dr. Rutherford has held the appointment of Health Officer for Houston and Harris county ten consecutive years, and to date.

In 1878, when yellow fever made its appearance at New Orleans, Dr. Rutherford, who was then Health Officer at Houston, received a letter from Dr. Ross, of Brenham, (who is said to have conceived the idea of centralizing the power of quarantine under one head), requesting him to call a meeting of all the health authorities of the various municipalities of the State for consultation. The call was responded to by a large number of acting health officers. After discussion, a centralized power was concluded to be the best plan, and Dr. R. was chosen unanimously to represent the views of the convention, and to carry out its idea. He was invested with authority to act for all. He accepted the trust, without fear or favor, and with no thought of remuneration, but with a certainty of much loss to his private practice; and his administration demonstrated the correctness of his views, that to be efficient, quarantine should be operated by one man, with plenary power, and the courage to act. Texas was spared an invasion of the pestilence that year. This convention and its fruits, it is said, lead directly to the passage of the present law, whereby the office of State Health Officer was created, and the unique system of quarantine now in operation. In 1879, after the passage of the act, Governor Roberts appointed Dr. Rutherford State Health Officer—the first appointee under the law. He served one term under Governor Roberts, and on the advent of the present administration, Gov. Ross again appointed him,—and the Senate confirmed the appointment on the 21st of February, 1887.

## Figures and Tables

**Figure f1:**